# Combining a Simple Method for DNA/RNA/Protein Co-Purification and Arabidopsis Protoplast Assay to Facilitate Viroid Research

**DOI:** 10.3390/v11040324

**Published:** 2019-04-03

**Authors:** Jian Jiang, Junfei Ma, Bin Liu, Ying Wang

**Affiliations:** Department of Biological Sciences, Mississippi State University, Starkville, MS 39762, USA; jiang@biology.msstate.edu (J.J.); jm5026@msstate.edu (J.M.)

**Keywords:** viroid, protoplast, DNA/RNA/protein co-purification

## Abstract

Plant–viroid interactions represent a valuable model for delineating structure–function relationships of noncoding RNAs. For various functional studies, it is desirable to minimize sample variations by using DNA, RNA, and proteins co-purified from the same samples. Currently, most of the co-purification protocols rely on TRI Reagent (Trizol as a common representative) and require protein precipitation and dissolving steps, which render difficulties in experimental handling and high-throughput analyses. Here, we established a simple and robust method to minimize the precipitation steps and yield ready-to-use RNA and protein in solutions. This method can be applied to samples in small quantities, such as protoplasts. Given the ease and the robustness of this new method, it will have broad applications in virology and other disciplines in molecular biology.

## 1. Introduction

The flow of genetic information from genome to transcriptome and then proteins dictates phenotypes in all organisms [1]. The genomic *cis*-regulatory elements and epigenetic hallmarks (e.g., cytosine methylation) in genomic DNA sequences pose transcriptional regulations over gene expression, the effects of which impact the corresponding mRNA and protein levels [2,3,4]. Comparison between mRNA and protein expression profiles can help uncover possible post-transcriptional [5,6,7] and/or translational controls [8,9]. These comparisons have been routinely performed in modern molecular biology to dissect gene functions.

It is notable that many genes exhibit rapid and sensitive responses to the surrounding environment, which makes collecting experimental materials from the same sample a necessity to ensure the maximum accuracy in subsequent analyses. Simultaneously extracting DNA, RNA, and proteins is possible through a guanidine isothiocyanate-based protocol [10], and several derivatives using TRI Reagent (containing guanidine isothiocyanate as a major component) have been developed with minor modifications [11,12,13,14,15,16]. Generally, TRI Reagent separates DNA, RNA, and proteins into different fractions. Sequential precipitation or a combination of such methodologies with specific nucleic acid-binding columns facilitate further purification. However, almost all such protocols require protein precipitation and dissolving steps, increasing difficulties in experimental handling and reducing yields. In addition, these protocols may not be as effective for valuable samples in small quantities, which further restrains their applications in research.

Here, we established a simple and robust method to co-purify DNA, RNA, and proteins from the same sample. This method does not employ TRI Reagent, has minimum precipitation steps, and generates ready-to-use RNA and protein solutions with high quality. Our analysis showed that both nuclear and cytosolic proteins with a wide-range of molecular weights can be effectively purified. Of note, this method is compatible with samples of small quantity.

We attempted to employ the powerful *Arabidopsis* genetic resources to facilitate studies on viroid biogenesis, using the protoplast replication system. Viroids are circular non-coding RNAs that mainly infect crop and ornamental plants [17,18,19]. Plant–viroid interactions represent a valuable model to delineate structure–function relationships of noncoding RNAs, which also has significant implications in multiple research areas in virology, plant biology, and molecular biology. However, the host machinery and the underlying mechanisms for viroid biogenesis are not yet fully understood due to technical challenges in biochemical approaches. Viroids cannot embark systemic infection in *A. thaliana* [18], which hindered the usage of *Arabidopsis* genetic resources for viroid research. Nevertheless, multiple viroids can replicate in *Arabidopsis* transgenic lines expressing their cDNAs, indicating the presence of conserved machinery for viroid biogenesis [20,21]. Here, we demonstrated the replication of potato spindle tuber viroid (PSTVd) in *Arabidopsis* protoplasts and successfully applied our method to co-purify DNA, RNA, and proteins. This experimental platform will significantly enhance our capacity to probe the host machinery and the functional mechanisms for PSTVd nuclear import/export and propagation. Moreover, our new method should have broad applications for various research in virology and other disciplines in molecular biology.

## 2. Materials and Methods

### 2.1. Plant Growth and Protoplast Assays

*Nicotiana benthamiana* plants were grown in a growth chamber at 25 °C and with a 16/8 h light/dark cycle. *A. thaliana* plants were grown in a growth chamber at 23 °C and with a 16/8 h light/dark cycle. Using 4-week old *Arabidopsis* plants, protoplasts were isolated following a published protocol [22]. Briefly, we used 3% cellulose (Onozuka Yakult Pharmaceutical IND., Tokyo, Japan) and 0.8% macerase (MilliporeSigma, Burlington, MA, USA) in digestion buffer (0.4 M mannitol, 20 mM KCl, 20 mM MES, 10 mM CaCl_2_, 0.1% BSA, 5 mM β-mercaptoethanol, pH 5.7) to digest leaves with the lower epidermis layer removed by tapes. After 1 h digestion, the protoplasts were pelleted using 1 min centrifugation at 150× *g*. The pelleted protoplasts were then sequentially incubated in the W5 buffer (5 mM MES, 154 mM NaCl, 125 mM CaCl_2_, 5 mM KCl, pH 5.7) on ice and MMg solution (4 mM MES, 0.4 M mannitol, 15 mM MgCl_2_, pH 5.7) at room temperature. About 10^5^ cells in 200 µL MMg buffer were supplemented with 40 μg *35S::*GFP plasmid and 5 µg RZ-Int PSTVd RNA [23], then mixed with 200 µL PEG solution (4 g PEG4000, 3.5 mL ddH_2_O, 2 mL 1 M mannitol, 1 mL 1M CaCl_2_) for 5 min incubation. Finally, protoplasts were washed with W5 solution and then incubated in WI solution (0.4 M mannitol, 20 mM KCl, 20 mM MES, pH 5.7) for 2 days before RNA purification. 

### 2.2. RNA Extraction and Analysis

Leaf samples were collected in 1.5 mL microcentrifuge tubes and ground in liquid nitrogen. The GeneJET plant RNA purification kit and the MagJET RNA Kit (Thermo Fisher Scientific, Waltham, MA, USA) were used for RNA purification as instructed in the manuals. Briefly, when using the GeneJET plant RNA purification kit, tissue lysate was loaded into the column for centrifugation. The flow-through (FL) was collected for subsequent protein purification. The column was washed twice using washing buffer from the kit and followed by DNase I treatment as instructed. Nuclease-free water was used to elute the total RNA from the column after the DNase I treatment. When using the MagJET kit, 40 µL of MagJET magnetic beads and 400 µL of ethanol (96–100%) were added to 400 µL tissue lysate free of tissue debris, and the mixture was kept rotating for 5 min at room temperature. The mixture was then placed in the magnetic rack for 1 min. The unbound supernatant fractions (Su) were collected for protein purification. The magnetic beads were then subjected to DNase I treatment and three washes. Finally, 100 µL of nuclease-free water was added to beads to elute total RNA. The flow-through solution collected after RNA binding to the GeneJET column and the supernatant solution collected after RNA binding to the MagJET beads were kept on ice for protein purification. The pellet of tissue debris from both purification kits were saved for genomic DNA extraction. For RNA purification from protoplasts, the cells were pelleted through 1000× *g* centrifugation for 2 min and then directly subjected to RNA purification using the MagJET RNA kit.

### 2.3. RT–PCR and RNA Gel Blots

For RNA analysis, the total RNA extracted from the above methods was subjected to reverse transcription (RT) using Superscript III reverse transcriptase (Thermo Fisher Scientific). We followed the manufacturer’s manual for first strand synthesis. The cDNA products were subjected to PCR using primers specific for GFP mRNA (16C f: 5′-ctcccacaacgtatacatcatggc-3′ and 16C r: 5′-ccatgccatgtgtaatcccagcag-3′). For RNA gel blots, we followed the protocol described previously in Reference [24]. Briefly, total RNA was electrophoresed on 5% (*w*/*v*) polyacrylamide/8 M urea gels for 1 h at 200 V, transferred to Hybond-XL nylon membranes (GE Healthcare Life Sciences, Pittsburgh, PA, USA) using a semi-dry transfer unit (Bio-Rad, Hercules, CA, USA), and immobilized by UV cross-linking. The membrane was blocked using Denhardt solution (VWR, Radnor, PA, USA) followed by overnight hybridization with digoxigenin (DIG)-labeled riboprobe at 65 °C. DIG-labeled PSTVd-specific probe was generated using *Hind*III-linearized pInt(−) plasmid [25] and a T7 polymerase MAXIscript kit (Thermo Fisher Scientific). Following the instructions of the DIG northern starter kit (Millipore Sigma), the membranes were washed and incubated with the antibody against DIG labeling. The alkaline phosphatase substrates were applied to the membranes, followed by fluorescence signal detection using C-DiGit (Li-COR Biosciences, Lincoln, NE, USA).

### 2.4. Protein Extraction

The FL fraction from the GeneJET kit and the Su fraction from the MagJET kit were subjected to protein purification using the Pierce SDS-PAGE Sample Prep Kit (Thermo Fisher Scientific). Briefly, the liquid fractions supplemented with DMSO were directly loaded into a spin column. The samples were subjected to centrifugation at 2000× *g* for 2 min. The flow-through was discarded. Columns were washed twice using the wash buffer and incubated with the elution buffer at 60 °C for 5 min, followed by centrifugation at 2000× *g* for 2 min to collect protein solutions. For RIPA buffer (radioimmunoprecipitation assay buffer) purification, leaves were ground to powders in liquid nitrogen and the powders (100 µL in volume) were directly mixed with 100 µL 1× RIPA (Millipore Sigma). The mixtures were incubated at 4 °C for 10 min followed by 14,000× *g* centrifugation at 4 °C for 3 min. The supernatants were used for analyses.

### 2.5. Silver Staining and Immunoblotting

The purified proteins were subjected to SDS-PAGE separation and transferred to nitrocellulose membranes using a semidry transfer unit (Bio-Rad). After 10 min incubation with Rapidblock solution (VWR), the following specific primary antibodies were applied for overnight incubation at 4 °C: anti-GFP (Genscript, Piscataway, NJ) 1:2000, anti-Histone H3 (Genscript) 1:2000 dilution, anti-beta tubulin (Genscript) 1:2000 dilution, the polyclonal antibodies (MilliporeSigma) against Mitogen-activated protein kinase 3 (MAPK3) 1:1000, the monoclonal antibody (8WG16; Thermo Fisher Scientific) against the largest subunit of RNA polymerase II (NRPB1) 1:500, and the polyclonal antibodies (Aviva Systems Biology, San Diego, CA) against ribosomal protein L5 (RPL5) 1:1000. After three washes with 1× TBST (19 mM Tris pH7.4, 137 mM NaCl, 2.7 mM KCl, 0.1% Tween 20), Horseradish peroxidase (HRP)-conjugated secondary antibody against rabbit (Millipore Sigma) was added at 1:3000 dilution for detecting Histone H3, RPL5, and MAPK3, while HRP-conjugated secondary antibody against mouse (Millipore Sigma) was added at 1:8000 dilution for GFP, beta tubulin, and NRPB1. After 1× TBST wash for three times and then incubation with HRP substrates (Li-COR Biosciences), the signals were captured with C-DiGit (Li-COR Biosciences). For silver staining, the gels after electrophoresis were treated with the Silver Bullit^TM^ kit (VWR), following instructions in the user manual.

### 2.6. DNA Extraction

The pellet of cell debris left from RNA purification was subjected to 1 mL DNAzol-ES following instructions from the vendor (Molecular research center Inc., Cincinnati, OH, USA). The mixture was subjected to centrifugation at 10,000× *g* for 10 min, and the supernatant was then transferred to a new centrifuge tube. Then 500 µL 100% ethanol was added to the supernatant and mixed at room temperature for 3 min. Genomic DNA was precipitated at 5000× *g* centrifugation for 5 min. The DNA pellet was washed twice with 1 mL ice cold 75% ethanol and air dried for 5 min. The pellet was dissolved using 20 µL TE buffer (10 mM Tris pH8.0, 1 mM EDTA). To clone the transcription factor IIIA (TFIIIA) fragment in *Arabidopsis*, two primers (AtTFIIIA genome p f: 5′-ggagacctcctgagaagctccagc-3′ and AtTFIIIA genome p r: 5′-gtccttatcacggttgtcattactatg-3′) were used. The PCR product was confirmed using agarose gel electrophoresis.

### 2.7. Southern Blots

About 20 µg of genomic DNA purified using our method or directly via DNAzol-ES was subjected to overnight digestion with *Nco*I and *Hind*III (New England Biolabs). The digested DNA was subjected to 1% agarose gel separation. After gel running, the agarose gel was subjected to depurination using 0.125 M HCl for 30 min, denatured (0.5 M NaOH and 1.5 M NaCl) for 30 min, and neutralized (0.5 M Tris pH7.5 and 1.5 M NaCl) for 30 min. The treated gel was subjected to transferring to the Hybond-XL membrane (GE Healthcare Life Sciences) using a vacuum transfer system (Stratagene, San Diego, CA, USA) and 20× SSC buffer (3 M NaCl and 0.3 M sodium citrate, pH7.2). The membrane was subjected to UV cross-linking, blocking, probe incubation, washing, and signal development using the DIG northern starter kit (MilliporeSigma). The GFP fragment cloned from genomic PCR (16C f: 5′-ctcccacaacgtatacatcatggc-3′ and 16C r: 5′-ccatgccatgtgtaatcccagcag-3′) was ligated into the pCR4 vector (Thermo Fisher Scientific) to generate the pCR4-GFP/probe. The pCR4-GFP/probe plasmid was digested using *Not*I (New England Biolabs) and served as a template for generating the DIG-labeled probe using the T3 Maxiscript kit (Thermo Fisher Scientific).

### 2.8. Bisulfite Sequencing

About 120 ng genomic DNA purified using our method was subjected to bisulfite treatment using the EZ DNA Methylation-Gold kit (Zymo Research, Irvine, CA, USA). The treated DNA was used as templates for PCR amplification using a modified pair of primers for GFP (Bisulfite 16C f: 5′- atgcYtgagggataYgtgcaggagagga-3′ and Bisulfite 16C r: 5′- ggacagggccatcRccaattggagtattttR-3′). The amplified product was ligated into the pCR4 vector (Thermo Fisher Scientific) and subjected to Sanger sequencing. The experiment was repeated twice.

## 3. Results

### 3.1. Recovering Proteins after RNA Enrichment

We chose transgenic *N. benthamiana* 16C plants with constitutive GFP expression as the test materials. A recent study showed that there is only a single copy insertion of GFP cDNA using the whole genome sequencing approach [26]. The total RNA was purified using the GeneJET plant RNA purification kit or the MagJET RNA purification kit, following manufacturer’s instructions. It is notable that the MagJET family has a variety of products to purify different RNA species, which is ideal for one-step enrichments of desired RNA populations for downstream analyses. As expected, we could easily detect GFP mRNA using total RNA purified from either the GeneJET plant RNA purification kit or the MagJET RNA kit by RT-PCR (Figure S1).

**Figure 1:**
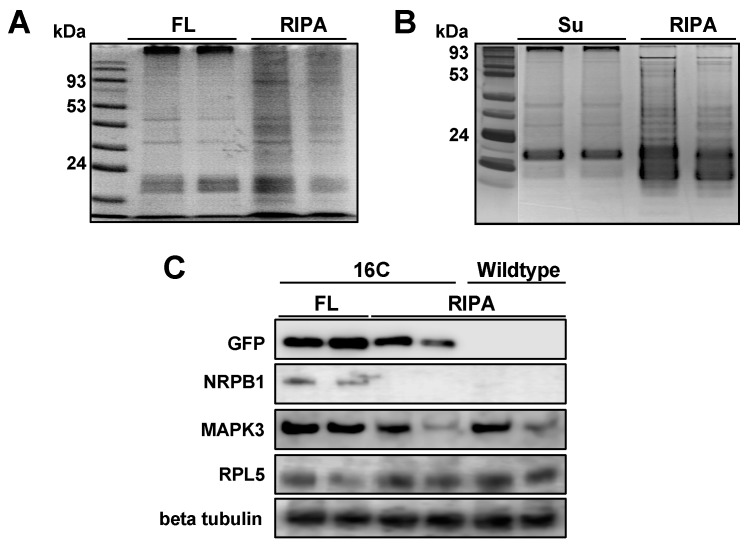
Silver staining and immunoblotting of purified proteins. (**A**) The comparison of proteins enriched from the flow-though (FL) using the GeneJET RNA purification kit with direct protein purification using radioimmunoprecipitation assay (RIPA) buffer; (**B**) the comparison of proteins enriched from the RNA-depleted supernatant (Su) using the MagJET RNA purification kit with direct protein purification using RIPA buffer; and (**C**) immunoblotting for comparing enriched proteins from our method and RIPA buffer by detecting GFP, NRPB1, Mitogen-activated protein kinase 3 (MAPK3), ribosomal protein L5 (RPL5), and beta tubulin. Protein size markers in (**A**,**B**) depict proteins sizes: 170, 125, 93, 72, 53, 42, 31, 24, and 15 (kDa). We only highlighted sizes 93, 53 and 24 (kDa) for illustration.

Because proteins normally pass through the column or remain in the supernatant after RNA binding to the magnetic beads, we reason that it is possible to recover proteins from the flow-through fraction (FL) after RNA binding to the GeneJET column or the unbound supernatant fraction (Su) after RNA binding to the MagJET beads. Interestingly, the SDS-PAGE Sample Prep Kit employs a DMSO-denaturing-based principle to enrich proteins and remove undesired chemicals from protein solutions. Therefore, we decided to test whether a combinational use of both RNA and protein purification kits can purify RNA and proteins from the same sample. We added DMSO to the FL and Su fractions and applied the mixtures to the SDS-PAGE Sample Prep Kit to enrich total proteins. After applying the enriched proteins to SDS-PAGE gel electrophoresis and silver staining, we observed effective recovery of total proteins from both FL and Su fractions. To evaluate the purification efficiency of our method, we chose the commonly used RIPA buffer purification for comparison. When comparing the recovering efficiency with proteins directly purified from leaf samples using RIPA buffer, recovery rates from FL and Su were lower (Figure 1A,B). Nevertheless, the major protein bands in RIPA buffer purified samples were present in samples prepared with our method. Furthermore, the consistent protein patterns in the replicates using our method indicates that this new method is reliable for protein analyses. As a further test, we performed immunoblotting assays and detected the presence of the GFP protein in recovered proteins from FL (Figure 1C) and Su (Figure S2) using our purification method. Interestingly, the GFP signals were stronger in proteins recovered from the FL fraction as compared with proteins directly purified using RIPA buffer (Figure 1C), likely due to the removal of undesired chemicals by the SDS-PAGE Sample Prep Kit. To further validate our method, we detected multiple endogenous proteins in recovered proteins from FL, including ribosomal protein L5 (RPL5; 35 kDa), beta tubulin (50 kDa), Mitogen-activated protein kinase 3 (MAPK3; 43 kDa), and the largest subunit of DNA-dependent RNA polymerase II (NRPB1; 220 kDa) (Figure 1C). It is noteworthy that these proteins vary in sizes (35–220 kDa) and subcellular localizations (e.g., the cytoplasm and nucleus), suggesting that our method is suitable for recovering a wide-range of proteins. Interestingly, our method is superior for detecting NRPB1 as compared with the RIPA buffer extraction (Figure 1C).

Since the Su fraction contains a high concentration of ethanol that can denature proteins, we tested whether proteins can be effectively recovered using the SDS-PAGE Sample Prep Kit without supplementing DMSO. As shown in Figure S2, silver staining of total proteins and immunoblotting for GFP demonstrated that omitting DMSO slightly reduced the protein recovery yield but still provided a desired recovery of proteins. Since supplementing DMSO into the RNA-depleted solution significantly increases the solution volume and centrifugation steps, it is possible to omit DMSO to improve the speed of purification process for high-throughput analyses.

### 3.2. DNA Recovery from Tissue Debris Using DNAzol-ES

Some studies require analysis of genomic DNA. Therefore, it is desirable to purify genomic DNA from the same sample together with the purification of RNA and proteins. We reasoned that tissue debris, leftover from the RNA purification steps, contained a sufficient amount of genomic DNA that could be purified and analyzed. We applied DNAzol-ES to the debris for genomic DNA purification. For Su fractions and their control replicates, six 16C seedlings (~1 mg) were pooled as the initial material for one replicate sample. In this case, genomic DNA was successfully recovered from the debris (157.5 ng and 192.5 ng in two replicates), and the recovery rate is about 5- to 7-fold less as compared with the direct DNAzol-ES purification (850 ng and 1090 ng in two control replicates). For FL fractions and their control replicates, we used ~10 mg leaf powder as the initial material for one replicate sample. In this case, genomic DNA recovery from the debris is a little more efficient (2.18 µg and 2.20 µg in two replicates), as the recovery rate is about 2.5-fold less than the direct DNAzol-ES purification (4.98 µg and 5.30 µg in control replicates). Although the DNA recovery is less in our new method as compared with direct DNA purification, it still provides a reasonable amount of genomic DNA for subsequent analyses. It is notable that if only genomic DNA and RNA are desired for analyses, a combinational use of RNAzol and DNAzol-ES as instructed in manuals will maximize the recovery of genomic DNA and RNA.

**Figure 2:**
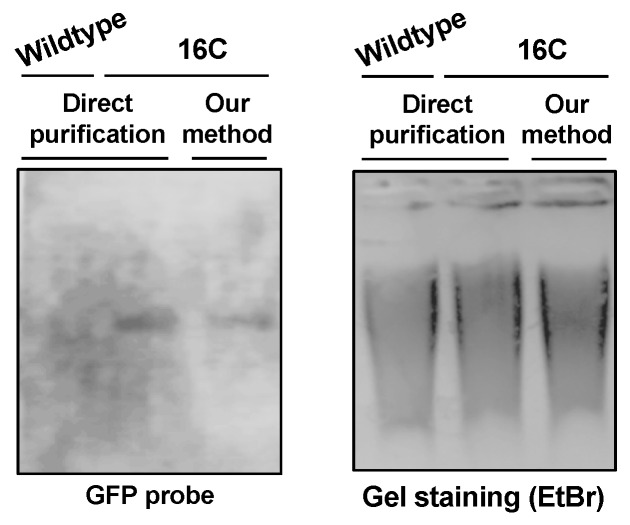
Southern blots for detecting GFP cDNA, with ethidium bromide staining used as the loading control.

**Figure 3:**
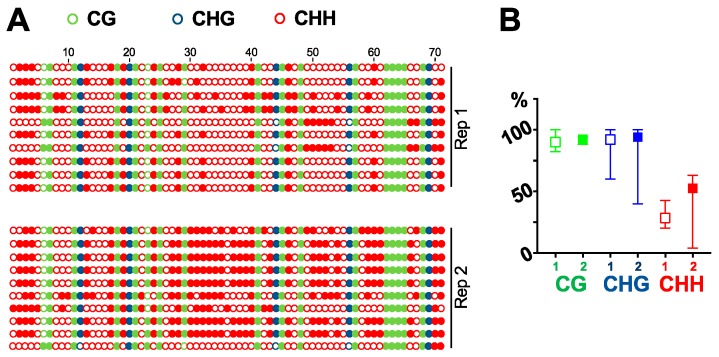
Bisulfite sequencing. Using bisulfite sequencing, we analyzed the cytosine methylations in a fragment of GFP cDNA in 16C plant genome. (**A**) All 71 cytosine residues are listed, with solid circles depicting methylated residues. (**B**) Frequency of methylated cytosine in CG, CHG and CHH. Replicate 1 is shown using an open-box symbol while replicate 2 is shown using a solid-box symbol.

DNA purified by DNAzol-ES is suitable for multiple downstream analysis, such as cloning, PCR, and southern blots. We can successfully clone the GFP fragment via PCR [27]. Here, we tested the purified DNA for southern blot analysis. Using a probe specific for GFP cDNA, we detected a single band in 16C genomic DNA but not in DNA from wildtype plants (Figure 2). We then tested whether the DNA purified by our method is suitable for bisulfite sequencing analysis. We treated the purified DNA to convert all the cytosine to uracil. If there was a methylation mark on a cytosine, the residue would remain without any change. We then amplified the region by PCR and sequenced ten clones for each of the two biological replicates to analyze the cytosine methylations in a GFP cDNA fragment in 16C genome. As shown in Figure 3, there are 17 CG, 5 CHG, and 49 CHH (where H equals to A, C, or T) in the analyzed region and almost all the cytosine in CG and CHG are methylated in both replicates. In contrast, methylation in CHH are more stochastic and less than half of them display methylation (Figure 3). This is in line with recent studies that report CG can be highly methylated but CHH is less methylated in actively transcribed gene bodies in plants [28,29]. This observation confirms that our method for genomic DNA purification captures the methylation marks and is suitable for DNA methylation studies.

**Figure 4:**
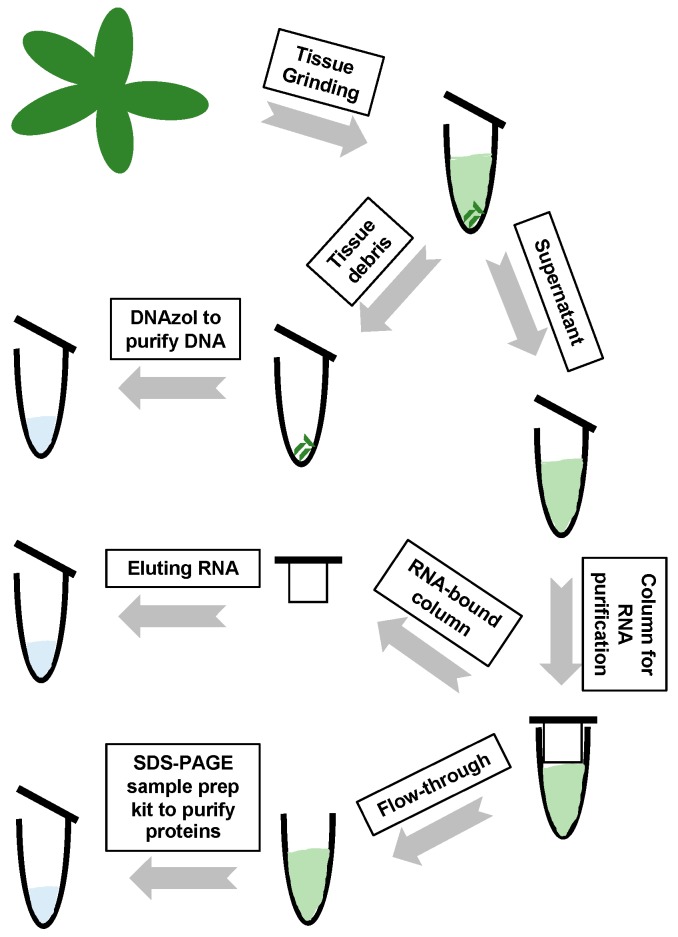
A flow chart demonstrating the procedures to co-purify DNA, RNA, and proteins.

With the successful recovery of genomic DNA from the same sample, we established an effective method to co-purify genomic DNA, RNA, and proteins from the same sample. As illustrated in Figure 4, samples are subjected to RNA purification by various commercial kits, and the flow-through fraction or the unbound supernatant are used for protein recovery using the SDS-PAGE Sample Prep Kit. Genomic DNA is recovered from tissue debris using DNAzol-ES.

### 3.3. Co-Purification of DNA/RNA/Protein from Protoplasts

**Figure 5:**
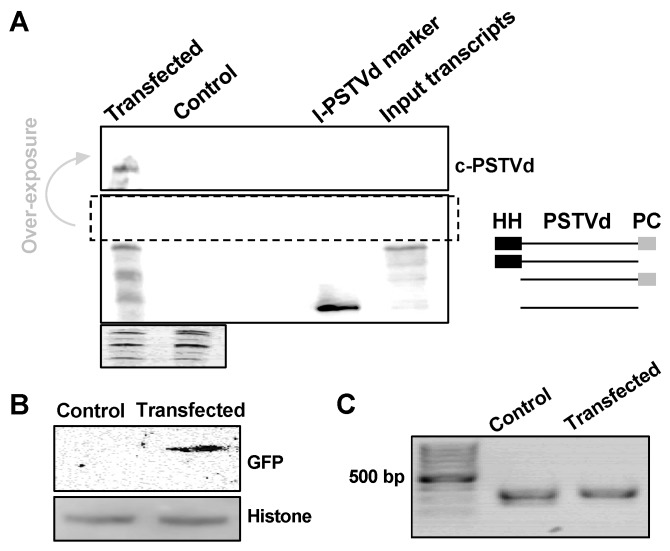
Co-purification of DNA, RNA, and proteins from protoplasts. PSTVd and *35S::*GFP co-transfected *Arabidopsis* protoplasts were harvested two days post-transfection. DNA, RNA, and proteins were co-purified using our new method. (**A**) RNA gel blotting detected the replication of PSTVd in *Arabidopsis* protoplasts, and the ethidium bromide staining of ribosomal RNAs served as a loading control. We used two size markers as shown in two right lanes on the blot: The unit-length linear PSTVd (l-PSTVd) RNA and the RZ-Int (input transcripts) RNA. The RZ-Int RNA contains a full-length RNA with a hammerhead ribozyme, an l-PSTVd, and a paperclip ribozyme arranged from the 5′ to 3′ end. The ribozyme activities result in four RNA species. The size illustration for the mixture of the RZ-Int inoculum is on the right panel. HH and PC represent the hammerhead and paperclip ribozymes, respectively. (**B**) Immunoblotting detected the expression of GFP, wherein histone H3 served as a loading control. (**C**) PCR using purified genomic DNA amplified a fragment from endogenous TFIIIA gene. c-PSTVd refers to the circular PSTVd genome.

We are interested in testing whether this method is efficient for small quantity samples, such as protoplasts. Here, we used RZ-Int PSTVd RNA as the inoculum, together with GFP reporter plasmids (35S::GFP), to co-transfect A. thaliana protoplasts. PSTVd replicates in A. thaliana [20,23] but cannot achieve systemic trafficking [20]. In our test, about 2 × 10^5^ protoplast cells were harvested two days post-transfection and were subjected to nucleic acids and protein purification using our method. As shown in Figure 5A, we could detect PSTVd replication using RNA gel blots, as indicated by the presence of circular genomic RNA. Immunoblots detected the expression of GFP, with Histone H3 as a loading control (Figure 5B). This also supports the notion that our method is suitable for small proteins (~15 kDa). Genomic PCR was successfully performed to amplify a fragment of the endogenous TFIIIA gene (Figure 5C). These results demonstrate that this new method is efficient for materials in small quantities. Importantly, these results also confirmed that our new method can effectively recover proteins from both cytosolic and nuclear compartments.

## 4. Discussion

Many genes exhibit rapid expression dynamics in response to diverse environmental stimuli, so using distinct components from the same sample will overtly enhance the accuracy of functional analyses. In this regard, methods to co-purify various biological components from the same sample are necessary. Currently, most of the available protocols to co-purify DNA, RNA, and proteins from the same sample are largely based on TRI Reagent and involve multiple precipitation steps [14]. These protocols all require precipitation of proteins and then dissolving of pellets, which is time-consuming and technically challenging. Here, by using the SDS-PAGE sample prep kit, we developed a method that can purify proteins after RNA purification by using commercial kits (column-based or magnetic bead-based). Genomic DNA can then be purified from tissue debris that are leftover from the initial RNA purification steps. 

This method is simple and straightforward and generates ready-to-use materials for downstream immunoblotting analysis, RNA gel electrophoresis/blots, regular cloning, and bisulfite sequencing. RNA was purified in the first step, which ensures the yield of high-quality RNA with minimum degradation while waiting. Of note, the DNA and RNA purification procedures are based on commonly used commercial kits, which have been routinely used to prepare RNA and DNA samples for RNA-Seq, degradomal RNA-Seq, and genomic sequencing. In this regard, our method is probably compatible for various deep sequencing technologies.

One advantage of our method is that it is compatible with small quantities of starting materials, such as protoplasts. Protoplast assays have been widely used in plant biology to rapidly and efficiently test: (1) The cellular localization of proteins and RNAs [30,31]; (2) plant responses to biotic stresses at transcriptional and post-transcriptional levels [32,33]; (3) protein–protein interactions [31,34]; (4) gene functions [35,36]; and (5) viral replications [25,37], etc. Due to limitations in sample quantities, current studies are often limited to microscopic analysis or to analysis of only one type of biological component (DNA, RNA, or proteins) in protoplasts, undermining the value of this transient transgenic approach. Our method will maximize the potential of *Arabidopsis* protoplast assays for functional studies.

Here, we would also like to point out that the combination of the *Arabidopsis* protoplast replication system and the co-purification method can potentially advance viroid research by opening the door to the powerful *Arabidopsis* genetic resources [38]. Viroids are circular noncoding RNAs that infect crop plants, often leading to plant disease [18,19]. As infectious noncoding RNAs, viroids have been a productive model to dissect plant defense mechanisms against foreign RNAs [23,39,40], the role of RNA three-dimensional motifs in regulating RNA systemic trafficking [18,41], and RNA-templated RNA replication by DNA-dependent RNA polymerase II [42,43]. Recent progress has begun to elucidate how viroids co-opt cellular factors to effectively propagate themselves [18,24,43,44,45,46,47,48,49,50,51,52,53,54,55,56]. In spite of these progresses, future studies are needed to unravel novel cellular factors involved in viroid biogenesis and their functional mechanisms. Using the *Arabidopsis* protoplast assay in combination with our co-purification method, viroid research can now take advantage of the potent genetic resource for future explorations. Our method presented here is also applicable to various other research in virology and molecular biology.

## Supplementary Materials

The following are available online at https://www.mdpi.com/1999-4915/11/4/324/s1, Figure S1: RT-PCR assessing RNA quality, Figure S2: Assessing the effect of DMSO.
