# Supplementary material for: Combining a Simple Method for DNA/RNA/Protein Co-Purification and Arabidopsis Protoplast Assay to Facilitate Viroid Research
Source: Viruses. 2019 Apr 3;11(4):324. doi: 10.3390/v11040324 (PMC6521142; doi:10.3390/v11040324)
Supplement: Supplementary file 1 [file viruses-11-00324-s001.pdf]

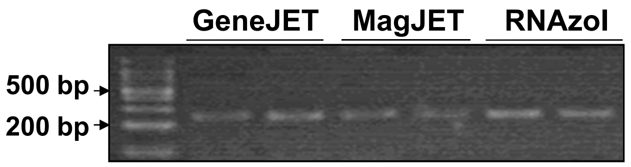

Figure S1. RT-PCR assessing RNA quality. RT-PCR of a *GFP* fragment confirms the successful purification of RNA.

Figure S1

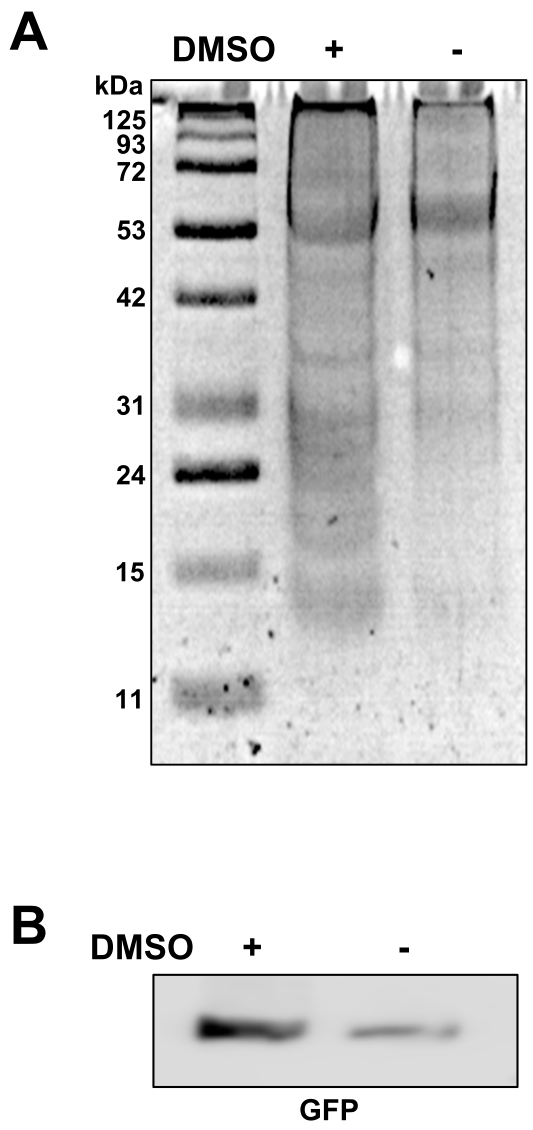

Figure S2. Assessing the effect of DMSO. Two equal volumes of RNA-depleted supernatant from MagJET RNA purification were treated with or without DMSO. Silver staining of SDS-PAGE gel (A) and immunoblotting (B) demonstrated that the supplementation of DMSO slightly enhanced the protein recovery rate, but may be omitted to simplify the protocol for high-throughput assays.

Figure S2
